# Antibiotic Resistant Superbugs: Assessment of the Interrelationship of Occurrence in Clinical Settings and Environmental Niches

**DOI:** 10.3390/molecules22010029

**Published:** 2016-12-27

**Authors:** Anthony Ayodeji Adegoke, Adekunle Christopher Faleye, Gulshan Singh, Thor Axel Stenström

**Affiliations:** 1 SARChI, Institute for Water and Wastewater Technology, Durban University of Technology, Durban 4000, South Africa; kunle_faleye@yahoo.co.uk (A.C.F.); gsingh.gulshan@gmail.com (G.S.); thors@dut.ac.za (T.A.S.); 2 Department of Microbiology, University of Uyo, 520211 Uyo, Akwa Ibom State, Nigeria; 3 Department of Biochemistry and Microbiology, University of Fort Hare, Alice 5700, Eastern Cape, South Africa

**Keywords:** residual antibiotics, antimicrobial resistance, total antibiotic resistance, critical control point, superbug, exposure, health risk assessment

## Abstract

The increasing threat to global health posed by antibiotic resistance remains of serious concern. Human health remains at higher risk due to several reported therapeutic failures to many life threatening drug resistant microbial infections. The resultant effects have been prolonged hospital stay, higher cost of alternative therapy, increased mortality, etc. This opinionated review considers the two main concerns in integrated human health risk assessment (i.e., residual antibiotics and antibiotic resistant genes) in various compartments of human environment, as well as clinical dynamics associated with the development and transfer of antibiotic resistance (AR). Contributions of quorum sensing, biofilms, enzyme production, and small colony variants in bacteria, among other factors in soil, water, animal farm and clinical settings were also considered. Every potential factor in environmental and clinical settings that brings about AR needs to be identified for the summative effects in overall resistance. There is a need to embrace coordinated multi-locational approaches and interrelationships to track the emergence of resistance in different niches in soil and water versus the hospital environment. The further integration with advocacy, legislation, enforcement, technological innovations and further research input and recourse to WHO guidelines on antibiotic policy would be advantageous towards addressing the emergence of antibiotic resistant superbugs.

## 1. Introduction

The emergence of antibiotic resistance (AR) is an outcome of a repertoire of factors in various environmental and clinical settings. Rizzo et al. [1] described wastewater treatment plants (WWTPs) as the hotspots for the emergence of AR. Additionally surface water and soil have equally been reported by several authors to harbour AR-inducing factors [1,2,3,4,5,6,7,8]. The critical role of the environment in the development and dissemination of antibiotic resistance genes (ARGs) is fast being appreciated, unlike the time when all focus was on hospital-acquired AR. Antibiotics and ARGs may partly originate from environmental bacteria, and selection for them is closely connected with anthropogenic contamination with residual antibiotics (RAbs), to which exposure of pathogenic bacteria in the same environment occurs [9]. Water environments are one of several platforms for the circulation and accumulation of discharged antibiotics and are recognized for dissemination of ARGs [10]. ARGs in water environments correlate with human activities that involve antibiotic usage [11,12,13]. The concentration of RAbs in the environment usually correlates with that of ARGs [14,15]. Antibiotic usage and/or abuse in human and veterinary medicine, and the pharmaceutical industries as well as their release with human and animal wastes are closely connected with increased prevalence of antimicrobial-resistant bacteria (AMRB) [16,17]. A sub-lethal concentration of antibiotic (or sub MIC) selects for resistant bacteria in any setting [18]. It is therefore imperative to harmonize various factors that bring about the exposure of bacteria to the sub-lethal or sub-inhibitory concentration of the antibiotics beyond the clinical settings, in line with WHO global action against AR [19]. Without any doubt, the origin and spread of antimicrobial resistance (AMR) is a very complex problem that is predicated by multifaceted contributing factors and requires multidisciplinary solutions [20,21]. This review will identify several factors in the environment involving wastewater, surface water, soil and important clinical or patient dependent factors and their interrelationship in contributing to AMR, as well as a possible novel approach to address it.

## 2. The Soil Resistome as a Contributor to AMR

Several antibiotics originate from bacteria and/or fungi from soil [2,22] as metabolites for outcompeting other organisms in the same niche, since competition among microorganisms is natural. From the history of antibiotics, the knowledge of these antagonistic interactions brought about the discovery of antibiotics [5,23,24]. This paved the way for the first major success in human struggle with infectious diseases, with an appreciable reduction in death worldwide [25,26]. No sooner was this success achieved than the observation of resistance to the first antibiotic, penicillin, was reported [27]. Similarly, the emergence of resistance has been repeatedly shown with each antibiotic subsequently discovered or developed [23].

Earlier, AR was defined in the light of increased MICs with no regard to microbial intrinsic ability to resist them [28] nor the potentials of the microbe to harbour ARGs, acquired vertically or horizontally. In addition the natural ability of antibiotic producers to resist their own metabolites (antibiotics) [5,29], it is now a known fact that various niches in the environment promote the emergence of AR as they contain pools of genes similar to ARGs in the hospital [30]. Antibiotic- producing bacteria which are known for resistance to their own antibiotics have inherent potentials or genes, some of which may be lost by them and gained by other bacteria in the same niche. The loss and gain in this respect may be supported by external stress in the soil microbiota. This is exemplified by the loss of plasmids by *Bacillus subtilis* due to interaction with *B. simplex* [31]. Various soil microbiota therefore are critical niches for the emergence and dissemination of ARGs with direct or indirect effect on the clinical isolates and therapeutic outcome on infectious diseases [6,7]. The soil thus becomes very significant because of reports of novel ARGs [30,31,32].

Factors enhancing the continued persistence of ARGs and AMR in soil include the use of organic fertilizers or manure containing RAbs, ARGs, AMRB, etc. [33]. The RAbs found in organic fertilizers originate from human activities. Organic fertilizer was in one instance shown to contribute as much as 20 mg·kg^−1^ of tetracycline to the soil, exposing the soil bacteria to sub-lethal concentrations with resistance as a consequence [34] and selection for antibiotic resistance genes which end up being released in the soil. The administration of the antibiotics for either therapeutic and prophylactic purposes in clinical settings as well as in livestock at <0.2 g·kg^−1^ to improve feed efficiency and growth rates [21,35] stands as the major contributing factor. The Alliance for Prudent Use of Antibiotics (APUA) emphasizes the huge impact of antibiotic administration in animal husbandry resulting in the overall rise in AR globally [36]. In America, up to 200,000 tons of antibiotics are used annually combined by humans and administered to farm animals [37]. This also includes antibiotic use in plant agriculture [38]. Table 1 provides a summary of the reported release of some RAbs as well as exemplifying some of the ARGs in soil, aquatic biomes and other related strata of the environment.

It is noteworthy that each time swine manure was applied to the soil, Pan et al. [39] reported that 0.001–29 mg·kg^−1^ of sulfonamides, 0.03–765 mg·kg^−1^ of tetracycline, and 0.05–0.11 mg·kg^−1^ macrolide were also released to the soil, as the manure contained them. This is closely linked to high induction of AR in soil, as well as the corresponding proportion of ARGs [40], making it critical to track and stop the emergence of resistance. The impact of emerging resistance in the soil would be appreciated by reconciling the deposit with the way microorganisms are recycled between hospital and environment. Despite this, the numbers of studies relating to tracking ARGs in the environment are limited. Most of the existing studies are limited to cultured microorganisms and with a focus on a few common resistance genes. Furthermore it is mainly guided by clinical reports with little or no reference to the environment. Accurate data on the ARGs as well as projection for potential AMRB in the environment could be acquired, if researchers designed such enumeration towards the uncultured microorganisms that constitute the majority in the environment [41].

Since ARGs reside in both cultured and uncultured bacteria, studies should consider research approaches using direct detection to determine the total ARGs in the environment. Similarly, more accurate detection of organisms exemplifies more accurate detection of total ARGs in the same microbiota. Several uncultured microorganisms harbour biotechnologically and public health important genes [23]. The use of direct detection (culture-free) techniques that account for uncultured bacteria in tracking the distribution of ARGs within the surface water environment is hereby advocated. As stated earlier, this will provide more effective insight into actual distribution of the total ARGs within the microbiota [17,23]. It also gives room for detection of novel ARGs in the study environment [17]. This is exemplified by Cheng et al. [17] reports that showed the usefulness of functional metagenomics in identifying new ARGs. Nesme et al. [30] studied antibiotic resistance in the environment more effectively through large-scale metagenomics-based investigations. The technique used enabled the author to describe diverse and abundant antibiotic resistance genes in nonclinical environments as well as to track their distribution. This was done without leaving the uncultured bacteria behind.

The soil may be a reservoir of larger pools of ARGs than ever imagined and various compartments like farmlands-water interphase, animal farm-surrounding interphase and sites where soil transport may occur due to rain run-off or animal movement may be critical for the occurrence of ARG and may be in focus for the assessment of their occurrence and further spread. Linking the environmental strains with clinic ones, promoted by human activities, might be a vital consideration [52,53,54]. In these cases, several factors like biofilm formation and quorum sensing (to be discussed later in this article) support the interaction.

## 3. RAbs and AMR by Aquatic Microbiota

Antibiotic concentrations in aquatic environments generally have been found to range from ng·L^−1^ to low μg·L^−1^ levels [3,4,8]. In a summary of studies reporting antibiotic concentrations in aquatic environments by Gros et al. [3], the median concentrations in surface and ground water were reported as 0.030 and 0.071 μg·L^−1^ respectively. Table 1 shows some examples of antibiotic concentrations in wastewater and hospital effluents. These concentrations are above the MIC/MBC for all the antibiotics, according to the British Society for Antimicrobial Chemotherapy [55]. The concentrations reduce due to photolytic effects as the RAbs are transported along the path of water flow and guarantees exposure to sub-MIC/sub-lethal concentration [40]. Gullberg et al. [18] demonstrated in vitro that resistant bacteria can be selected for, at antibiotic concentrations lower than the MIC. The half-life varies. For oxytetracycline, for example, it depends on the prevailing conditions in the environment, such as temperature, light intensity and flow rate. For the quinolone oxolinic acid, a mean half-life (photolytic) of 298 days and 509 days in light and dark conditions, respectively, has been reported [56]. The countries listed in Table 1 have low capacities to remove antibiotic contaminants, although improvements have occurred in the conventional WWTPs (e.g., AAO) [57] that are mainly in use.

The concentrations in vitro are different from the ones in the wastewater environment, since the RAbs may form complexes with other chemicals or adsorb onto particulates. The complexes so formed may cause more ecotoxic effects and induce more broad range resilience (resistance) in the bacteria than the original RAbs. Some of these RAbs can further induce the transfer of ARGs and recombinate in aquatic bacteria, at sub-lethal concentrations [58,59], suggesting that exchange of ARGs may be common in environments contaminated with antibiotics.

Assessments of the discharge from the manufacturing companies have shown that extremely high concentrations of antibiotics in wastewater have been closely linked to antibiotic-manufacturing industrial effluents [14]. The concentration decreases with dilution and photolytic effects in the wastewater treatment and in the receiving environment. The original OTC residues of 920 ± 20 mg·L^−1^ were reduced to 30.5 ± 1.1 mg·L^−1^ when mixed and later to 19.5 ± 2.9 mg·L^−1^ in the wastewater effluent. The OTC concentration in the receiving river decreased to 264 μg·L^−1^ (probably by photo-degradation) at 20 km from the discharge point [47]. This explains the critical impact of industrial effluents (especially from pharmaceutical companies) on the discharge of RAbs and the emergence of resistance. Li et al. [14] further showed that 94.2% bacterial isolates in wastewater and 95.4% in receiving water bodies harboured 67.0% Tet(A). They also harboured Tet(W), Tet(C), Tet(J), Tet(L), Tet(D), Tet(Y), and Tet(K) ranging between 21.0% and 40.6%. The Tet genes might have been acquired vertically by the microorganisms or emerged by selection as adaptation for survival [60].

Emergence of resistance through bacterial exposure to RAbs in aquatic microbiota depends also on the time of exposure. Li et al. [14] observed that bacterial isolates in downstream parts (after the point of discharge for wastewater effluents) of rivers exhibit higher multiple antibiotic resistance index (MARI) and harbour more ARGs than those upstream of the effluent point. The author ascribed this to the impact of wastewater treatment plants as there was a correlation in the observed MAR Index/ARGs between the wastewater and the river studied [14]. Another potential contributing factor is longer exposure time as the bacteria remained exposed to the RAbs present in the water along the path of flow [60]. ARGs residing on plasmids and integrons are also shared when bacteria are together for a longer time [61,62]. More research is hereby advocated for to determine the role of uncultured bacteria in the ARGs surge as reported by Zhang et al. [63]. Meanwhile, pertinent studies have identified ARGs now isolated from treated water as well as among bacteria from freshwater but previously believed to be in exclusive reserve of clinically important bacteria [64,65,66,67]. There is further need for studies tracking the source of such genes in aquatic environments accounting for different potential sources of contamination.

## 4. Emergence of AMR in Clinical and Sub-Clinical Settings

**Figure 1:**
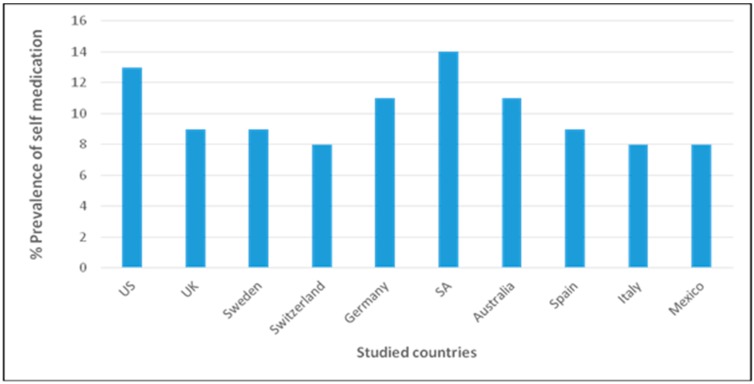
Percentage self-medication in some developed and mid-economic developed countries (Adapted from: http://www.abimip.org.br/uploads/material_de_ apoio/1296056417_792.pdf/).

The emergence of AMR in clinical settings considers the contribution from human medicine and its impact on hospital-environmental interface, as well as outpatient administration of antibiotics, leading to exposure of individuals to sub-lethal concentrations of antibiotics. Several non-environmental factors have been reported to predicate AMR in clinical settings. However, some factors are underrated, yet they contribute into the alarming rate of AMR emergence in hospitals. The consequence encompasses therapeutic failure, prolonged hospital stay, higher cost of alternative treatment option and possibly higher risk of death [68]. In developing countries where the control on antimicrobial drugs (AMD) administration is weak and antibiotics are sold like over-the-counter (OTC) drugs, individuals with symptoms suspected to be similar to an infectious outcome engage in self-medication prior hospital visit [69]. Self-medication remains a current challenge towards the emergence of AMR [70]. Self-medication is not limited to developing countries. It extends to countries with strict control on drug sales and administration [71]. Figure 1 gives an overview of percentage prevalence of self-medication in developed and middle economic countries. Countries like Switzerland and Italy have lower prevalence of self-medication (8%) as compared to South Africa (Figure 1), potentially due to sale restrictions. Orally administered AMDs are more subjected to self-medication than parenteral as the former ones are self-administered; in overused and underused regimen, leading to several contraindications, as well as the emergence of AMR [72].

Poor audit system of the prescriptions in many countries has encouraged the prescription of antibiotics without reference to laboratory analysis. Inappropriate prescription is in no way better than self-medication as both promote AMR [72,73]. Between 30% and 50% inappropriate prescription reflected by wrong indication, incorrect choices of AMD and regimen have been reported [73,74]. These are also closely related to patients’ non-compliance to a correct regimen prescribed by physician, the two aberrations lead the failure to achieve and maintain the required plasma concentrations (inhibitory or lethal dose) within the period of drug administration.

Administration of counterfeit drugs also contributes extensively to the emergence of antibiotic resistance in developing countries. Factors contributing to fake or substandard drugs which are common in developing countries include the economic incentives and weak intellectual property protection in those countries. Counterfeit drugs include drugs without active ingredients, drugs with lower concentrations of active ingredients than required and drugs with different ingredients from the indicated label. Drugs with lower concentration of active ingredients are likely to induce antibiotic resistance [75], as bacteria get exposed to sub-lethal concentrations of the drug in vivo and become adapted to them.

## 5. Reported Emerging Threat Level of AMR

As emphasized, emergence of resistance is induced by exposure to RAbs in the soil, wastewater and in hospital environment, contributing to rise in the level of threat to public health. The Centers for Disease Control and Prevention [74] identified the following selected organisms as serious threats especially in the United States: pan-drug resistant (PDR) or extended spectrum drug resistant (XDR) *Acinetobacter* spp., drug-resistant *Campylobacter* spp., fluconazole-resistant *Candida* spp., extended spectrum β-lactamase-producing *Enterobacteriaceae* (ESBLs), vancomycin-resistant Enterococci (VRE), multidrug-resistant *Pseudomonas aeruginosa*, drug-resistant Non-typhoidal *Salmonella* spp., drug-resistant *Salmonella*, methicillin-resistant *Staphylococcus aureus* (MRSA), drug-resistant *Streptococcus pneumoniae*, total drug-resistant *Mycobacterium tuberculosis*, etc. The threat associated with these selected microorganisms was described by Senekal [76] as being important in the emerging order of AR. They are therefore further described in Table 2 based on the report of other researchers.

These organisms are able to effectively develop resistance in clinical settings. In the environment individuals may for example be exposed to them through aerosols from hospital effluents treated in WWTPs. This unfortunately is worsened by the presence of antibiotics in sub-inhibitory concentrations [142]. *Chromobacterium violaceum* ATCC 12472 was reported by Liu et al. [144] to form antibiotic-induced quorum sensing and biofilm formation which lead to exchange of genetic information and acquisition of resistance in aquatic and terrestrial habitat. Quorum sensing (QS) occurs following the production, detection and cells’ response to small diffusible signal molecules called auto-inducers. The molecules determine the type of quorum formed. The possible quorum sensing can be:

a LuxI/LuxR–type quorum sensing: The signal molecules utilized here are the acyl-homoserine lactones (AHL) and they are found in Gram-negative bacteria, for example the complex QS machinery in *Acinetobacter* is mediated by LuxI/LuxR system peculiar to Gram-negative bacteria. This cell signalling system is made up of AHL [145].
b Oligopeptide-bicomponental quorum sensing: This utilizes small peptides as signal molecules and are found in Gram-positive bacteria.

Therefore, whichever molecule would be produced viz-a-viz the quorum formed depends on the bacterial types as well as the inducing factor (s), but they all affect the biofilm production and the accessibility of the organism to the antibiotics. The procedure for the formation of biofilm is the same in both clinic and the environment.

Meanwhile, the mechanisms associated with biofilm for antibiotic resistance is both innate and induced [146,147]. This leads to reduction in the concentration of antibiotics that reach the bacterial cells and their environment [148]. Certain antibiotics like ampicillin penetrate only selected biofilms like those formed by *Klebsiella pneumoniae* with no potential for β-lactamase production, but not stable enough to the enzyme to penetrate those formed by wild type with ability to produce the hydrolase [149], so large populations of cells are harboured in biofilms where they undergo genetic exchange leading to antibiotic resistance [150]. Even in human body fluid, bacteria produce biofilms to shield themselves from antibiotics, selective pressure, as well as from opsonin-phagocytosis [151,152,153]. This is mediated by accessory gene regulator (agr)-mediated quorum sensing in *Staphylococci* [152]. Beside biofilms, Table 3 further states some other associated attributes and mechanisms that contribute to the emergence of resistance in the bacterial reservoirs.

**Figure 2:**
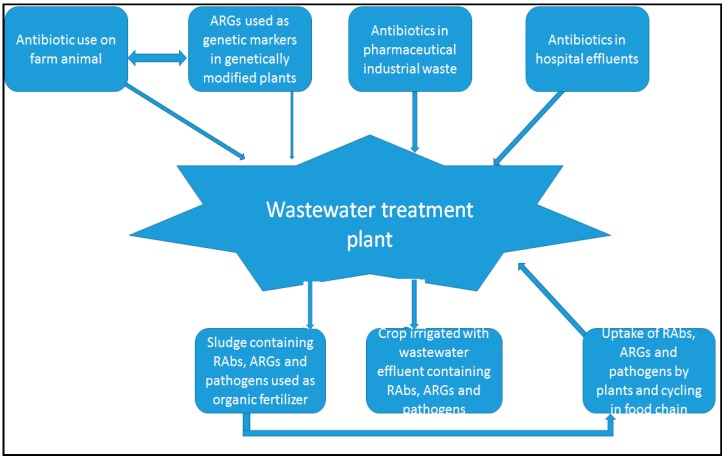
The interrelatedness of contributing factors to AMR (AMR Microbes, RAbs and ARGs are recycled in nature).

The importance of mutations cannot be overemphasized. Recovery from exposure to sub-inhibitory or sub-lethal concentration of antibiotics may lead to adaptation via mutation. The evolution of AMR under the sub-MIC arises progressively as low-cost mutations (e.g., duplications and amplifications) in high frequencies [155]. This continues in each reservoir where bacteria are exposed to sub-inhibitory concentration of the indicated antibiotics. The trend may be worsened, if the organism(s) is conveyed from one reservoir to another, exposing them to wider range of antibiotics. This is why coordinated efforts in all compartments of the environment are imperative in checkmating the emergence of AMR. Figure 2 clearly depicts the links in various compartment of human environment and how the RAbs, emerging AMR bacteria already getting exposed to RAbs, ARGs, etc. are conveyed in cycles. This long interrelated cycle of exposure and emergence of AMR probably begat the present level of threat and should be addressed at each stage, but in a coordinated manner.

## 6. Coordinated Approaches towards Addressing the Emergence and Spread of AMR

Coordinated multi-locational approaches across various segment of the environment should be embraced towards preventing the emergence or spread of AMR and its uprising threat. The global action plan against antimicrobial resistance suggested the outright prevention of any infection or those caused by multiple antibiotic resistant bacteria (MARB) appears as the most likely pathway to follow. This can be achieved directly by personal and general hygiene. There is also an indirect pathway to prevent community (environmentally)-acquired infections by ensuring proper treatment of wastewater. This reduces the associated risks of infection to the user of the effluents and the residents around the receiving water environment. It should be noted that pathogens and RAbs in improperly treated wastewater effluents for reuse in irrigation are reportedly deposited on the surfaces or internalized in fruits and vegetables [33]. Consumption of MARB in uncooked foods like salads poses the greatest risks to the consumers [160,161]. This also applies to the reuse of the improperly treated sludge as organic fertilizer, exposing the farmers and crop consumers to the risks of MARB and difficult-to-treat infections [33]. The spread of MARB through wastewater treatment plants (WWTPs) or sludge deposition to other segments of the environment needs to be controlled and proper treatment applied. The compliance to this is however low, especially in developing economies. It is a settled fact that very low (sub-inhibitory) concentration of antibiotics reaches the river catchments. This may sustain continued development of resistance to antibiotics via exposure. WWTPs have also been identified as potential control points in an early warning system [15,47], especially those receiving wastewater from hospitals, which will be hotspot for MARB [1,51]. The two main concerns in integrated human health risk assessment associated with the development and transfer of antibiotic resistance in the environment (i.e., RAbs and ARGs) should therefore be considered in remodelling the WWTPs [162]. Technologies to remove RAbs, ARGs and MARB from wastewater may include membrane filtration, activated carbon, photo-driven nano-technologies and ozonation [163].

It is also imperative to channel more research towards in-depth understanding of the molecular, evolutionary and ecological mechanisms of AMR. Advocatory, legislation and enforcement on the control of drug abuse, restricting the use of antibiotics in agriculture exclusively to therapeutics and enforcing hospitals as well as pharmaceutical companies to own their specially configured WWTPs are pertinent steps. Relatively, the development of more antibiotic regimens in one daily dose (OD) e.g., using gastrointestinal float delivery technology as in Zanocin OD, a brand of ofloxacin [164] will improve patient compliance and reduce the emergence of AMR.

It is the right time to promote more intensive screening for new antimicrobial drug producing bacteria, development of new antibiotics and antibiotic targets as well as developing new diagnostics. Adegoke (unpublished data) discovered a strain of *Streptomyces* sp. Anthony DS-7A (http://www.uniprot.org/taxonomy/1827503) with far outstanding antimicrobial activity than erythromycin. Similarly, Ling et al. [165] detected a new antibiotic that kills pathogens without detectable resistance. Some of the potential sources of antimicrobial drugs remain unutilized. Palonbo [166] also remarked that despite several reports of high antimicrobial activity against MARB exhibited by plant extracts, very negligible numbers have been promoted to clinical stages. Novel antibiotics targeting the virulent factors and AMR inducing factors like quorum quenching to interfere with quorum sensing and biofilm formation have been solicited [167,168]. Such potent antibiotics should be able to hack into the bacterial biofilm [169], break its edges and ensure penetration of inhibitory concentration of its active ingredients to attack shielded bacteria.

## 7. Conclusions

Coordinated approaches to reduce integrated human health risks in the environment as well as careful compliance with the WHO guidelines [170] on surveillance, rational antibiotic prescribing, standard treatment guidelines for both community- and hospital-acquired infections will lead appreciably towards reducing the ever-rising threat of antibiotic resistance. Strategic steps related to assessment and management in various environmental reservoirs and niches will assert collective reduction in the threat and prevent the emergence of more aggressive superbugs.

## Figures and Tables

**Table 1:** Reported sub-lethal concentrations of residual antibiotics (RAbs) and antibiotic resistance genes (ARGs) in soil, aquatic environments and other related strata of the environment.

Environment	Source	RAb/ARGs	Reported Concentration	Country	References
Soil	Soil	CIP	2.77 μg/kg	Pakistan	[42]
		OFL	2.98 μg/kg		
		LEV	3.35 μg/kg		
		OXT	4.53 μg/kg		
		DOX	3.12 μg/kg		
	Grape soil	*Sul 1*	(39.19 ± 0.77) × 10^−2^	China	[43]
		*sulII*	(0.42 ± 0.08) × 10^−3^		
		*sulIII*	(0.48 ± 0.10) × 10^−3^		
		*tetA*	(0.02 ± 0.00) × 10^−3^		
		*tetB*	(0.44 ± 0.07) × 10^−3^		
		*tetO*	(10.55 ± 1.23) × 10^−2^		
	Soil	SMT	0.01 μg/g	China	[43]
		OTC	0.02 μg/g		
	Vegetable soil	TET	8400 μg/kg	China	[44]
	Animal manure	BAC	0.01–1.76 mg/kg	Canada	[45]
Aquatic Environment	Wastewater	CIP	3.0–5.25 mg/L	Pakistan	[42]
		LEV	0–6.20 mg/L		
		OFL	2.45–4.12 mg/L		
		OTC	0–9.40 mg/L		
		DOX	1.58–6.75 mg/L		
		AMX	6.94 μg/L	Australia	[46]
		CIP	0.72 μg/L	Hong Kong	[47]
		OFL	0.60 μg/L	Italy	[48]
		ERY	2.5–6.0 μg/L	Germany	[49]
	Surface water	OFL	0.31 μg/L	Italy	[48,50]
	Hospital effluents	AMX	35.12 μg/L	Brazil	[51]
		AMP	389.13 μg/L		
		CFX	300.1 μg/L		
		PEN G	434.46 μg/L		

Key: AMX = Amoxicillin; BAC = Bacitracin; CFX = Cefotaxime; CIP = Ciprofloxacin; DOX = Doxytetracycline; ERY = Erythromycin; LEV = Levofloxacin; OTC = Oxytetracycline; OFL = Ofloxacin; PEN G = Penicillin G; SMT = Sulphamethaxazole; TET = Tetracycline.

**Table 2:** Some reported AMR classified as having serious threat.

Bacteria Threat Level	Examples of Reported Antibiotics/Antibiotic Groups to Which Resistance Occurred	Countries Where This Has Been Reported	References
Pan drug resistant (PDR)/Extended spectrum drug resistant (XDR) *Acinetobacter* spp.	Resistant to at least 3 classes + Carbapenems, polymyxins, tigecycline or fluoroquinolones	Greece, US, India, South Africa, Iran, Greece	[77,78,79,80,81,82]
Drug resistant *Campylobacter* spp.	Range of 45% to 94.7% resistant to Erythromycin, azithromycin, clindamycin, telithromycin, ciprofloxacin,	US; Finland; Poland; Philippines; China; Nigeria	[83,84,85,86,87]
Fluconazole-resistant *Candida* spp.	8.0%–98.8% resistant to Itraconazole, voriconazole, caspofungin, echinocandin, amphotericin B deoxycholate, fluconazole	US, UK, Argentina, Spain, China, South Africa	[88,89,90,91,92]
Extended spectrum β-lactamase producing Enterobacteriaceae (ESBLs)	23% to 85.1% resistant to cephalosporins, gentamicin, kanamycin, streptomycin, nalidixic acid, ciprofloxacin, tetracycline, chloramphen-icol, sulfamethoxazole	US, Switzerland, Netherland, Saudi Arabia, France, Germany, Czech Republic, Sweden	[93,94,95,96,97,98,99,100,101,102]
Vancomycin-resistant *Enterococcus* (VRE)	≤90% ampicillin, chloramphen-icol, clindamycin, ciproflo-xin, erythromycin, neomycin, penicillin, rifampicin, tetracycline and vancomycin	US, Spain, Portugal Sweden, UK, Australia, Iran, Ethiopia	[103,104,105,106]
Multidrug-resistant *Pseudomonas aeruginosa*	20% to 85.7% Cefepime, piperacillin-tazobactam, piperacillin, amikacin, levofloxacin, ciprofloxacin, Ofloxacin, meropenem, etc.	US, India, Germany South African, Nigeria, Greece	[107,108,109,110,111,112]
Drug-resistant Non-typhoidal *Salmonella* spp.	≤100% resistant to nalidixic acid, tetracycline, streptomycin, ciprofloxacin, azithromycin and cefotaxime	US, Iran, Egypt, Ethiopia, UK, China, Congo Republic, Saudi Arabia, Greece	[113,114,115,116,117,118,119,120]
Drug-resistant *Salmonella*	Resistant to ceftriaxone, cefuroxime, amoxicillin, ampicillin, ciprofloxacin and augmentin	US, Nigeria, India, Southern Asia and Kenya	[121,122,123,124,125]
Methicillin-resistant *Staphylococcus aureus* (MRSA)	Usually resistant to wide range of beta lactam antibiotics to ≤100%	US, Nigeria, South Africa, Tanzania, several countries in Europe	[126,127,128,129,130,131]
Drug-resistant *Streptococcus pneumoniae*	e.g., 37% were resistant to erythromycin, 29.6% to cefotaxime, 7.4% to levofloxacin, and 14.8% were identified as multidrug resistant	US, Spain, India, Austria Belgium, France, Germany, Italy, Portugal, Spain and Switzerland	[132,133,134,135,136,137,138]
Total Drug-resistant *Mycobacterium tuberculosis*	>30 cases of TDR-TB reported. 32% of patients with MDR-TB exhibited resistance to a fluoroquinolone	India, Iran, Italy and South Africa	[139,140,141,142,143]

**Table 3:** Bacterial attributes besides selection for ARGs that facilitates AMR.

Attributes/Mechanism	Application/Example (s)	Reference (s)
Quorum sensing	Mediated by accessory gene regulator (agr)	[154]
Biofilm formation	Increased interaction of high population densities and close distant cells in biofilms for genetic exchange among mixed microbial communities converting biofilms to hotspots for antibiotic resistance GacS-GacA system is associated with the production of small-colony variants that affect motility, biofilm formation, and antibiotic resistance	[150,155,156]
Enzyme production	Beta lactamases, extended spectrum beta lactamase, metallo beta lactamase, etc. induced by exposure to imipenem and piperacillin in *P. aeruginosa* biofilms	[157]
Mutation	The evolution of AMR under the sub-MIC arises progressively as low-cost mutations (e.g., duplications and amplifications) in high frequency (Canton and Morosini, 2011)	[155]
Small colony variant (SMV)	Down-regulation of the bacterial electron transport and/or dihydrofolate reductase (DHFR) pathway sulfamethoxazole resistance, bringing about small colonial form GacS-GacA system	[156,157,158]
Target change	C1 metabolism e.g., Trimethoprim, Sulfamethoxazole, Daptomycin, Colistin, Gentamicin, streptomycin, spectinomycin etc.	[159]
